# Physiological Role of Serum Growth Differentiation Factor-15 (GDF-15) Level and Iron Metabolism in Community-Dwelling Older Adults

**DOI:** 10.7759/cureus.60422

**Published:** 2024-05-16

**Authors:** Taira Fukuda, Hiroko Yazawa, Riichi Nishikawa, Seiko Tokoi, Ryo Kayashima, Kaori Kono, Masashi Sakuma, Shichiro Abe, Shigeru Toyoda, Toshiaki Nakajima

**Affiliations:** 1 Department of Liberal Arts and Sciences, Kanagawa University of Human Services, Yokosuka, JPN; 2 Department of Cardiovascular Medicine, Dokkyo Medical University School of Medicine, Mibu, JPN; 3 Department of Fundamental Nursing, Dokkyo Medical University School of Nursing, Mibu, JPN; 4 Department of Medical KAATSU Training, Dokkyo Medical University School of Medicine, Mibu, JPN

**Keywords:** skeletal muscle mass index, community-dwelling older adults, hepcidin, growth differentiation factor-15, iron

## Abstract

Background

Anemia is common in older adults and, together with heart failure and chronic kidney disease, forms a vicious cycle, whereas diseases such as chronic inflammation and cancer are associated with the anemia of chronic disease (ACD). Researchers have linked growth differentiation factor-15 (GDF-15) to a variety of conditions such as cardiovascular disease, inflammation, cancer, and kidney disease, and have reported hepcidin as a biomarker for iron regulation in ACD. Therefore, anemia, GDF-15, and hepcidin have significance in aging physiology.

Hypothesis

GDF-15 and hepcidin play important physiological roles in community-dwelling older adults. This study sought to explore the relationship between these biomarkers and anemia, inflammation, or other health outcomes.

Methods

This was a prospective study of 73 community-dwelling older adults (six men and 67 women, mean age of 76.3 years). Their serum iron level, percentage transferrin saturation (TSAT), high-sensitivity C-reactive protein (hs-CRP), and estimated glomerular filtration rate (eGFR) were measured. Enzyme-linked immunosorbent assays were used to assess their serum GDF-15, ferritin, and hepcidin levels. The participants’ grip strength and walking speed were measured. The skeletal muscle mass index (SMI) of each participant was determined by bioelectrical impedance analysis.

Results

The GDF-15 level was significantly inversely correlated with serum iron, ferritin, and hepcidin levels; percentage TSAT; the eGFR; and gait speed. Serum hepcidin was positively correlated with levels of ferritin, albumin, and hemoglobin. Handgrip strength, SMI, and hs-CRP were not correlated with either GDF-15 or hepcidin levels. After adjusting for age, sex, and body mass index (BMI), multivariate analysis identified the log GDF-15 and serum iron level (log GDF-15: β=-0.248, iron: β=0.296) as significant factors determining hemoglobin levels, whose findings have significance due to novel results. Multivariate analysis identified eGFR and levels of hemoglobin and hepcidin as significant factors associated with log GDF-15 (eGFR: β=-0.406, hemoglobin: β=-0.269, hepcidin: β=-0.235). Similarly, ferritin and albumin levels were identified as significant factors associated with hepcidin levels (ferritin: β=0.590, Alb: β=0.277).

Conclusions

Anemia in community-dwelling older adults was determined not only by increasing serum iron levels but also by decreasing GDF-15 levels. Also, the increasing GDF-15 level was determined by a decreasing hepcidin level as well as the presence of anemia and renal dysfunction, and the decreasing hepcidin level was determined by decreasing stored iron and decreasing albumin levels. Serum GDF-15 and hepcidin could potentially inform diagnostic or treatment strategies for anemia or age-related health conditions.

## Introduction

Prevalence and impact of anemia in older adults

Anemia is very common in older adults and is associated with physical and cognitive impairment [1]. Hepcidin is a regulator of iron metabolism, and growth differentiation factor-15 (GDF-15) is a biomarker for anemia. Chronic inflammation and related conditions, such as cancer and autoimmune diseases, lead to "the anemia of chronic disease (ACD)”. Studies focusing on the regulation of iron homeostasis have identified two key factors in ACD: hepcidin and ferroportin (FPN) [2]; the activity of the latter is downregulated by hepcidin. [3,4].

Relationship between anemia, heart failure, and chronic kidney diseases

Heart failure, chronic kidney disease (CKD), and anemia also form a vicious cycle, with anemia causing and exacerbating heart failure, which leads to increased hospitalization and mortality rates and also induces malnutrition. Heart failure leads to the progression of CKD, and anemia induces deterioration in renal function [5].

Role of inflammation and hepcidin in the anemia of chronic disease

Hepcidin, a small peptide hormone produced in the liver, is a recently identified hormone related to iron metabolism. It may be a useful biomarker for many disorders [6]. By using hepcidin as a biomarker, we may have a tool to directly report the role of iron regulation. Hepcidin measurements may also reveal the underlying causes of treatment-resistant iron deficiency. The most common cause of anemia is iron deficiency, but it does not always respond to iron supplementation therapy. Bregman et al. [7] reported that elevated hepcidin levels are often associated with supplementation-resistant anemia. There are also pathological conditions (cancer, inflammation, and neuropathy) for which hepcidin measurements may have clinical relevance [8]. A potential practical diagnostic use for the measurement of hepcidin concentrations is the identification of patients with various conditions associated with altered iron metabolism.

Significance of GDF-15 in anemia and iron metabolism

We recently showed that GDF-15 is a determinant of hemoglobin (Hb) level in community-dwelling older adults [9]. Very high levels of GDF-15 have been reported to suppress hepcidin expression [10]. In contrast, moderately elevated GDF-15 levels were not significantly correlated with hepcidin expression in older patients with anemia of unknown origin [11]. Thus, the association between GDF-15 and hepcidin expression remains unknown. In patients with heart failure, GDF-15 was also associated with creatinine levels, red blood cell count, Hb levels, hepcidin, and total iron binding capacity. It tended to be associated with ejection fraction (EF) in univariate analysis, while hepcidin, creatinine, and EF were GDF-15 determinants in multivariate analysis [12]. However, the associations between markers of iron metabolism such as GDF-15 and hepcidin in community-dwelling older adults remain unclear.

The purpose of this study was to investigate the relationships between serum levels of GDF-15, hepcidin, and iron and their associations with Hb levels in community-dwelling older adults. This study investigated the associations between Hb levels and serum GDF-15, hepcidin, renal function, and muscle function in community-dwelling older adults.

## Materials and methods

Participants

The study was conducted at Kanagawa University of Human Services, Yokosuka, Japan. A total of 73 community-dwelling older adults (age: 76.3 ± 6.6 years; six men (8%), and 67 women (92%)) were enrolled. All participants lived in Yokohama and Yokosuka City and regularly attended an exercise class at Kanagawa University of Human Services. None of the participants had serious conditions. Participants were required to have a certain level of physical function or health status to participate in the exercise class. The study was approved by the Ethics Committee of the Kanagawa University of Human Services (No. 7-20-37) and the Regional Ethics Committee of Dokkyo Medical University Hospital (No. 27074). Informed consent was obtained from all participants.

Handgrip strength for the right hand was measured twice, and a higher value was used. Gait speed was measured as the time needed to walk 10 m. Two measurements were taken, and a higher value was adopted. Blood samples were collected after a 12-hour fast. Hb, serum iron, transferrin saturation (TSAT), ferritin, and serum albumin (Alb) levels were measured. The high-sensitivity C-reactive protein (hs-CRP) level was measured via an immunonephelometric assay (BML Inc., Tokyo, Japan). The estimated glomerular filtration rate (eGFR) was evaluated with the use of the following equation:

eGFR (mL/min/1.73m2) = 194 × serum creatinine-1.094 × age-0.287 (men)

eGFR (mL/min/1.73m2) = 194 × serum creatinine-1.094 × age-0.287 × 0.739 (women)

Enzyme-linked immunosorbent assay (ELISA)

To measure fasting serum GDF-15, ferritin, and hepcidin concentrations, peripheral venous blood was collected in pyrogen-free glass test tubes with and without ethylenediaminetetraacetic acid (EDTA) in the morning. Plasma and serum samples for all ELISAs were stored at -80 °C. The serum GDF-15 level was measured with the Human Quantikine ELISA Kit (DGD150 for GDF-15; R&D Systems, Minneapolis, MN, USA) as previously reported [13]. The mean intra-assay coefficient of variation (CV) was 2.3%, and the inter-assay CV was 5.4%. The detection threshold for GDF-15 was 0.002 ng/mL. The serum ferritin level was measured with the Human Ferritin ELISA Kit (ab108698; Abcam plc., Abcam, Cambridge, CB2 0AX, UK). The mean intra-assay CV was <5.4% and the inter-assay CV was <6.1%. The detection threshold for ferritin was 0.53 ng/mL. The serum hepcidin level was measured with the Human Hepcidin Quantikine ELISA Kit (DHP250; R&D Systems, Minneapolis, MN, USA). The mean intra-assay CV was <4.3% and the inter-assay CV was <11%. The detection threshold for hepcidin was 1.7 pg/mL. Samples, reagents, and buffers were prepared according to the manufacturer’s recommendations. No samples were outside the detection range of the assay, and no additional dilutions were made for use in the ELISA.

Bioelectrical impedance analyzer (BIA) measurements

The multi-frequency bioelectrical impedance analyzer (BIA), an InBody S10 Biospace device (Biospace Co., Ltd., Korea/Model JMW140), was used according to the manufacturer’s guidelines. BIA estimates body composition using the difference in conductivity of the various tissues based on the differences in their biological characteristics. Conductivity is proportional to water content and, more specifically, to electrolytes, and it decreases as the cell approaches a perfect spherical shape. Adipose tissue is composed of round-shaped cells and contains relatively little water compared to other tissues like muscle; therefore, conductivity is decreased as body fat increases. In practice, electrodes are placed at eight precise tactile points of the body to achieve a multi-segmental frequency analysis. A total of 30 impedance measurements were obtained using six different frequencies (1, 5, 50, 250, 500, and 1000 kHz) at the five following segments of the body: the right and left arms, trunk, and right and left legs [14]. Body fat volume, body fat percentage, and skeletal muscle mass were calculated. The skeletal muscle mass index (SMI) was calculated as (appendicular skeletal muscle mass, kg)/(body height, m)2. Sarcopenia was evaluated and defined according to the Asian Working Group for Sarcopenia (AWGS) criteria (men: handgrip strength <26 kg or walking speed ≤0.8 m/s, and SMI <7.0 kg/m2; women: handgrip strength <18 kg or walking speed ≤0.8 m/s, and SMI <5.7 kg/m2) [15].

Statistical analysis

All data are presented as means ± standard deviation, medians and interquartile ranges, or numbers and proportions depending on their distributions. Missing values in Hb (n=1), SMI (n=1), and gait speed (n=2) were excluded. After testing normality by the Kolmogorov-Smirnov method, associations between parameters were evaluated by the Pearson method for normally distributed parameters and by the Spearman method for non-normally distributed parameters. Univariate linear regression analyses with Hb level, log (serum GDF-15 concentration), and hepcidin as the dependent variables were performed to identify independent influencing factors (e.g., clinical laboratory or physical data). Age, sex, and body mass index (BMI) were used as covariates. Multivariate linear regression analysis was performed to identify the independent influencing factors in the five highest standardized partial regression coefficients (β) value parameters of the univariate analyses, except for TSAT. IBM SPSS Statistics for Windows, Version 28 (Released 2021; IBM Corp., Armonk, New York, United States) was used for statistical analysis. A p-value of <0.05 was regarded as significant.

## Results

Characteristics of the study participants

The mean age of the participant population was 76.3 ± 6.4 years, and the mean BMI was 22.7 ± 2.8 kg/m2. The mean handgrip strength was 23.6 ± 4.9 kg, and the median gait speed was 1.38 (1.22-1.55) m/s. The median SMI was 6.00 (5.70-6.48) kg/m2. The mean eGFR was 67.3 ± 13.1 mL/min/1.73 m2, and the mean Hb level was 13.1 ± 1.2 g/dL. The median serum Alb level was 4.3 (4.2-4.5) g/dL, and the median hs-CRP level was 0.04 (0.02-0.11) mg/L. The mean serum iron level was 91.1 ± 28.5 μg/dL, and the mean TSAT was 24.8 ± 8.0%. The mean serum ferritin level was 99.8 ± 59.5 ng/mL, and the mean serum hepcidin level was 24.1 ± 15.4 ng/mL. The median serum GDF-15 level was 0.91 (0.72-1.17) ng/mL. Among the participants, four (6%) had sarcopenia. The mean values for Hb, Alb, or eGFR fall within the expected ranges for older adults, and there were no notable deviations from typical values. Values for physical and clinical laboratory tests are summarized in Table 1. Sarcopenia is the loss of muscle mass with age, as determined by muscle functions such as handgrip strength, gait speed, and SMI, and the muscle functions and hs-CRP, indicative of inflammation, were within the normal range for older adults.

**Table 1:** Participants’ parameters Data are shown as means ± standard deviation, medians and interquartile range, or numbers and proportion. BMI: body mass index; SMI: skeletal muscle mass index; eGFR: estimated glomerular filtration rate; Hb: hemoglobin; Alb: albumin; hs-CRP: high-sensitivity C-reactive protein; TSAT: transferrin saturation; GDF: growth differentiation factor

	Total (n=73)
Age, years	76.3 ± 6.4
Sex (male/female)	6 (8%)/ 67 (92%)
BMI, kg/m^2^	22.7 ± 2.8
Handgrip strength, kg	23.6 ± 4.9
Gait speed, m/s	1.38 (1.22-1.55)
SMI, kg/m^2^	6.00 (5.70-6.48)
eGFR, mL/min/1.73m^2^	67.3 ± 13.1
Hb, g/dL	13.1 ± 1.2
Alb, g/dL	4.3 (4.2-4.5)
hs-CRP, mg/L	0.04 (0.02-0.11)
Iron, μg/dL	91.1 ± 28.5
TSAT, %	24.8 ± 8.0
Ferritin, ng/mL	99.8 ± 59.5
Hepcidin, ng/mL	24.1 ± 15.4
GDF-15, ng/mL	0.91 (0.72-1.17)

Correlation between clinical parameters and serum concentrations of GDF-15, hepcidin, and iron

Table 2 shows the relationships between the serum GDF-15, hepcidin, and iron levels, as well as the relationships of each of those parameters with the other parameters measured in the participants. The serum GDF-15 level was positively correlated with age (r≕0.445, p<0.001) and inversely correlated with sex (r≕-0.269, p=0.022) and gait speed (r≕-0.373, p=0.001). The correlations between GDF-15 levels, handgrip strength, and SMI were not significant. The serum GDF-15 level was inversely correlated with the eGFR (r≕-0.454, p<0.001) and serum iron (r≕-0.302, p=0.009), TSAT (r≕-0.334, p=0.004), ferritin (r≕-0.236, p=0.044), and hepcidin (r≕-0.348, p=0.003) levels. The serum hepcidin level was positively correlated with Alb (r≕0.344, p=0.003), Hb (r≕0.308, p=0.009), and ferritin (r≕0.615, p<0.001) levels. The serum iron level was positively correlated with serum Alb (r≕0.271, p=0.020), Hb (r≕0.473, p<0.001), TSAT (r≕0.905, p<0.001), and ferritin (r≕0.250, p=0.033) levels (Table 2). Serum GDF-15 was positively correlated with age and inversely correlated with eGFR and serum ferritin. Thus, serum GDF-15 was associated with aging, decreased renal function, and decreased iron stores.

**Table 2:** Correlations of participants’ parameters with serum GDF-15, hepcidin, and iron Data are shown as r-values (p-values). * p<0.05, ** p<0.01, *** p<0.001. + for positive correlations, - for negative correlations. GDF: growth differentiation factor; BMI: body mass index; SMI: skeletal muscle mass index; eGFR: estimated glomerular filtration rate; Alb: albumin; Hb: hemoglobin; hs-CRP: high-sensitivity C-reactive protein; TSAT: transferrin saturation

	GDF-15	Hepcidin	Iron
Age	+0.445 (<0.001)***	-0.135 (0.254)	-0.059 (0.620)
Sex	-0.269 (0.022)*	+0.192 (0.104)	+0.143 (0.227)
BMI	+0.031 (0.794)	+0.176 (0.137)	+0.144 (0.225)
Handgrip strength	-0.034 (0.774)	+0.068 (0.568)	-0.031 (0.791)
Gait speed	-0.373 (0.001)**	+0.155 (0.196)	+0.086 (0.476)
SMI	-0.083 (0.487)	+0.138 (0.247)	+0.172 (0.149)
eGFR	-0.454 (<0.001)***	+0.074 (0.536)	+0.076 (0.523)
Alb	-0.147 (0.215)	+0.344 (0.003)**	+0.271 (0.020)*
Hb	-0.172 (0.149)	+0.308 (0.009)**	+0.473 (<0.001)***
hs-CRP	+0.144 (0.225)	+0.083 (0.483)	-0.224 (0.057)
Iron	-0.302 (0.009)**	+0.153 (0.197)	-
TSAT	-0.334 (0.004)**	+0.184 (0.119)	+0.905 (<0.001)***
Ferritin	-0.236 (0.044)*	+0.615 (<0.001)***	+0.250 (0.033)*
Hepcidin	-0.348 (0.003)**	-	+0.153 (0.197)
GDF-15	-	-0.348 (0.003)**	-0.302 (0.009)**

Multivariate linear regression analysis of hemoglobin, serum GDF-15, and hepcidin levels with clinical parameters

Table 3 shows the β and p values from the multivariate regression analysis of hemoglobin in relation to clinical parameters. A linear regression analysis of Hb as the dependent variable and clinical data (ferritin, hepcidin, log GDF-15, iron, TSAT, eGFR, handgrip strength, gait speed, SMI, serum Alb, and log hs-CRP) as independent variables were conducted for all study participants. After adjustments for age, sex, and BMI, the univariate analysis found that ferritin (β=0.339, p=0.003), hepcidin (β=0.261, p=0.022), log GDF-15 (β=-0.441, p<0.001), iron (β=0.464, p<0.001), TSAT (β=0.459, p<0.001), gait speed (β=0.273, p=0.045), and Alb (β=0.260, p=0.024) were independent variables in relation to the Hb level. After adjustments for age, sex, and BMI, multivariate analysis found that the log GDF-15 (β=-0.248, p=0.032) and iron (β=0.296, p=0.006) were independent variables in relation to the Hb level (Table 3). Multivariate regression analysis showed that Hb was not only positively correlated with serum iron but also inversely correlated with serum GDF-15. Thus, in community-dwelling older adults, anemia was determined not only with low serum iron but also with high serum GDF-15 levels.

**Table 3:** Multivariate linear regression analysis of hemoglobin and iron metabolism, renal function, and muscle functions Model 1: univariate analysis adjusted by age, sex, and BMI; Model 2: multivariate analysis, unadjusted; Model 3: multivariate analysis adjusted by age, sex, and BMI. Data are shown as β-values (p-values). * p<0.05, ** p<0.01, *** p<0.001. GDF: growth differentiation factor; Hb: hemoglobin; TSAT: transferrin saturation; eGFR: estimated glomerular filtration rate; SMI: skeletal muscle mass index; Alb: albumin; hs-CRP: high-sensitivity C-reactive protein; BMI: body mass index

Dependent variable: Hb			
	Model 1	Model 2	Model 3
Adjusted R^2^		0.274	0.408
Independent variable	β-value (p)	β-value (p)	β-value (p)
Ferritin	0.339 (0.003)**	0.229 (0.090)	0.233 (0.060)
Hepcidin	0.261 (0.022)*	0.047 (0.738)	-0.029 (0.818)
GDF-15 (log)	-0.441 (<0.001)***	-0.098 (0.391)	-0.248 (0.032)*
Iron	0.464 (<0.001)***	0.341 (0.004)**	0.296 (0.006)**
TSAT	0.459 (<0.001)***		
eGFR	0.121 (0.305)		
Handgrip strength	0.217 (0.114)		
Gait speed	0.273 (0.045)*		
SMI	0.123 (0.442)		
Alb	0.260 (0.024)*	0.120 (0.285)	0.159 (0.129)
hs-CRP (log)	0.029 (0.818)		

Table 4 shows the β and p values from the multivariate regression analysis of serum GDF-15 levels in relation to clinical parameters. A linear regression analysis of log GDF-15 as the dependent variable and clinical data (ferritin, hepcidin, iron, TSAT, eGFR, handgrip strength, gait speed, SMI, serum Alb, Hb, and log hs-CRP) as the independent variable was conducted for all study participants. After adjustments for age, sex, and BMI, univariate analysis found that hepcidin (β=-0.266, p=0.016), Fe (β=-0.363, p=0.001), TSAT (β=-0.354, p=0.001), eGFR (β=-0.442, p<0.001), and Hb (β=-0.431, p<0.001) were independent variables in relation to the log GDF-15. After adjustments for age, sex, and BMI, multivariate analysis found that hepcidin (β=-0.235, p=0.038), eGFR (β=-0.406, p<0.001), and Hb (β=-0.269, p=0.020) were independent variables in relation to the log GDF-15 (Table 4). Thus, serum GDF-15 was determined not only by anemia and impaired renal function but also by decreased serum hepcidin levels.

**Table 4:** Multivariate linear regression analysis of GDF-15 and iron metabolism, renal function, and muscle functions Model 1: univariate analysis adjusted by age, sex, and BMI; Model 2: multivariate analysis, unadjusted; Model 3: multivariate analysis adjusted by age, sex, and BMI. Data are shown as β-values (p-values). * p<0.05, ** p<0.01, *** p<0.001. GDF: growth differentiation factor; Hb: hemoglobin; TSAT: transferrin saturation; eGFR: estimated glomerular filtration rate; SMI: skeletal muscle mass index; Alb: albumin; hs-CRP: high-sensitivity C-reactive protein; BMI: body mass index

Dependent variable: log GDF-15			
	Model 1	Model 2	Model 3
Adjusted R^2^		0.349	0.469
Independent variable	β-value (p)	β-value (p)	β-value (p)
Ferritin	-0.203 (0.071)	0.126 (0.329)	0.166 (0.162)
Hepcidin	-0.266 (0.016)*	-0.259 (0.038)*	-0.235 (0.038)*
Iron	-0.363 (0.001)**	-0.284 (0.012)*	-0.197 (0.058)
TSAT	-0.354 (0.001)*		
eGFR	-0.442 (<0.001)***	-0.435 (<0.001)***	-0.406 (<0.001)***
Handgrip strength	-0.118 (0.371)		
Gait speed	-0.097 (0.469)		
SMI	-0.190 (0.215)		
Alb	-0.140 (0.222)		
Hb	-0.431 (<0.001)***	-0.125 (0.281)	-0.269 (0.020)*
hs-CRP (log)	0.079 (0.527)		

Table 5 shows the β and p values from the multivariate regression analysis of serum hepcidin levels in relation to clinical parameters. A linear regression analysis of the serum hepcidin level as the dependent variable and clinical data (ferritin, log GDF-15, iron, TSAT, eGFR, handgrip strength, gait speed, SMI, serum Alb, Hb, and log hs-CRP) as independent variables was conducted for all study participants. After adjustments for age, sex, and BMI, the univariate analysis found that ferritin (β=0.602, p<0.001), log GDF-15 (β=-0.310, p=0.016), Alb (β=0.331, p=0.006), and Hb (β=0.292, p=0.022) were independent variables in relation to the serum hepcidin level. After adjustments for age, sex, and BMI, multivariate analysis found that ferritin (β=0.590, p<0.001) and Alb (β=0.277, p=0.007) were independent variables in relation to the serum hepcidin level (Table 5). Thus, serum hepcidin levels were determined by high serum albumin levels and high stored iron levels.

**Table 5:** Multivariate linear regression analysis of hepcidin and iron metabolism, renal function, and muscle functions Model 1: univariate analysis adjusted by age, sex, and BMI; Model 2: multivariate analysis, unadjusted; Model 3: multivariate analysis adjusted by age, sex, and BMI. Data are shown as β-values (p-values). * p<0.05, ** p<0.01, *** p<0.001. GDF: growth differentiation factor; Hb: hemoglobin; TSAT: transferrin saturation; eGFR: estimated glomerular filtration rate; SMI: skeletal muscle mass index; Alb: albumin; hs-CRP: high-sensitivity C-reactive protein; BMI: body mass index

Dependent variable: hepcidin			
	Model 1	Model 2	Model 3
Adjusted R^2^		0.430	0.421
Independent variable	β-value (p)	β-value (p)	β-value (p)
Ferritin	0.602 (<0.001)***	0.579 (<0.001)***	0.590 (<0.001)***
GDF-15 (log)	-0.310 (0.016)*	-0.141 (0.168)	-0.168 (0.143)
Iron	0.121 (0.320)		
TSAT	0.155 (0.199)		
eGFR	0.099 (0.426)		
Handgrip strength	0.098 (0.492)		
Gait speed	0.247 (0.086)	-0.052 (0.613)	-0.044 (0.727)
SMI	-0.063 (0.704)		
Alb	0.331 (0.006)**	0.247 (0.012)*	0.277 (0.007)**
Hb	0.292 (0.022)*	-0.017 (0.874)	-0.068 (0.575)
hs-CRP (log)	0.115 (0.391)		

**Figure 1:**
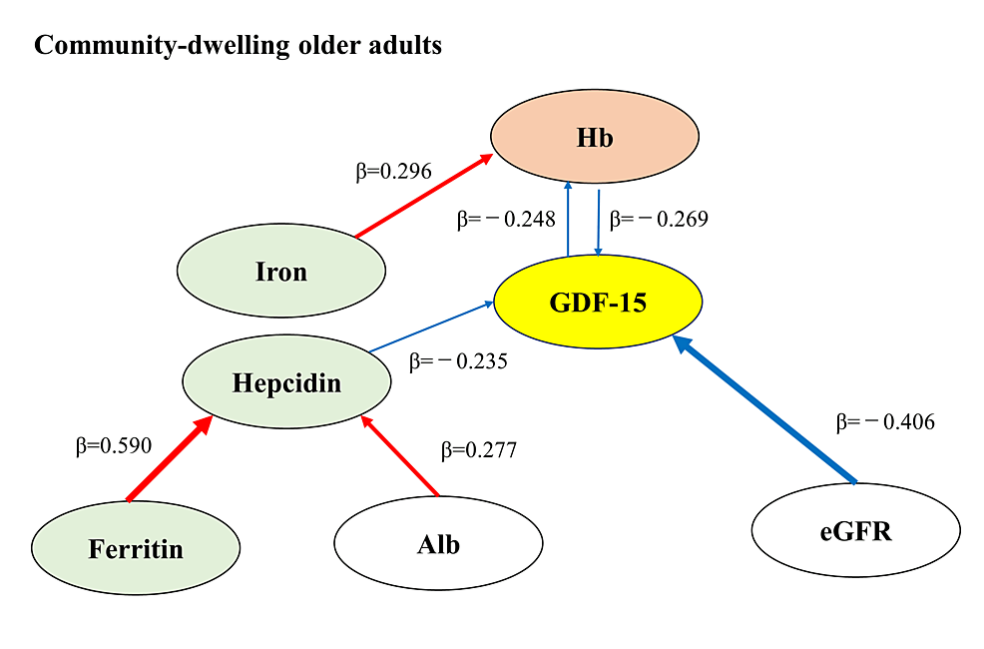
Graphic abstract Red lines show a positive correlation and blue lines show a negative correlation.

A graphic abstract showing the relationship of each parameter is presented in Figure 1.

## Discussion

In this study of community-dwelling older adults, GDF-15 was found to be inversely correlated with the serum iron, TSAT, ferritin, and hepcidin levels; eGFR; and gait speed. The serum hepcidin level was positively correlated with ferritin, Alb, and Hb. Handgrip strength, SMI, and hs-CRP were not significantly correlated with either GDF-15 or hepcidin. After adjustments for age, sex, and BMI, multivariate analysis identified the log GDF-15 and the serum iron level as factors related to the Hb level (log GDF-15: negative correlation; iron: positive correlation). The eGFR, Hb, and hepcidin levels were identified as factors related to the log GDF-15 (all inversely correlated), and ferritin and Alb were identified as factors related to the hepcidin level (all positively correlated).

Biological pathways through which GDF-15 and hepcidin influence iron metabolism and erythropoiesis

Hepcidin is a hormone produced in the liver that plays a central role in the regulation of iron metabolism in the body, suppressing the serum iron level through intestinal iron absorption, iron supply from stored and tissue iron, and iron recycling from the reticular system. The production of hepcidin is regulated by multiple factors, including iron saturation in the body, inflammation, and hematopoietic status [2]. Hepcidin binds directly to its receptor, the iron channel ferroportin (FPN), and enters the cells of the duodenum, liver, and reticular system. Once in the cell, FPN is degraded by lysosomes via ubiquitination [3,16]. This reaction reduces the expression of FPN in the cell and inhibits the translocation of iron to the extracellular space. FPN expression is highly regulated by transcription factors, the availability of heme, iron, and other metals (zinc and copper), and post-transcriptionally through a well-defined iron regulatory protein (IRP)/iron-responsive element (IRE) system [17].

Interleukins (ILs) such as IL-6 are elevated in infections and chronic inflammatory diseases [18,19], and all these pathological conditions are accompanied by increased hepcidin expression through activation of the Janus kinase (JAK)/signal transducer and activator of transcription (STAT) 3 intracellular signaling pathway. Hepcidin production is also induced by IL-6 during infection, which leads to the transient inhibition of subsequent FPN-mediated iron transport, resulting in decreased serum iron levels. In ACD, the persistent decrease in FPN due to high hepcidin levels reduces the absorption of dietary iron and the recycling of iron, leading to decreased levels of available iron. In this study of community-dwelling older adults, the serum GDF-15 and hepcidin levels were not correlated with the hs-CRP level, and hs-CRP was not a determinant of their levels.

Relevance of GDF-15 and hepcidin for the management and treatment of conditions such as anemia and cardiovascular disease in older adults

In patients with heart failure, those with a ferritin level ≥300 ng/mL and serum iron ≤13 μmol/L had decreased Hb levels and increased mortality rates [20,21]. Low serum iron and high iron stores reflected more severe heart failure than low serum iron and low iron stores due to cardiac dysfunction [22], and heart failure may be a key stimulus for inflammation and the secretion of hepcidin. Also, increased hepcidin production led to decreased iron absorption, increased ferritin levels, and increased mortality in patients with ACD [23]. In our study of community-dwelling older adults, ferritin, a marker of iron stores, was identified as a determinant of hepcidin levels.

GDF-15 is a determinant of mortality and has been reported to be useful in clinical practice [24,25]. Age-dependent GDF-15 percentiles in healthy participants were independent determinants of all-cause mortality, and GDF-15 reference values were a useful tool for risk stratification in clinical practice [24]. GDF-15 was also a powerful determinant of overall mortality and cardiovascular and non-cardiovascular mortality and could be a valuable addition to the traditional risk factors, N-terminal pro-brain natriuretic peptide and C-reactive protein (CRP), that are used for community-dwelling older adults [25].

In this study, multivariate regression analysis revealed that after adjustments for age, sex, and BMI, serum iron and log GDF-15 were factors determining the Hb levels. A recent study of patients with CKD that examined the association between anemia, GDF-15, and hepcidin found significant correlations between hepcidin levels and iron and CRP and significant correlations between serum GDF-15 and Hb levels, ferritin, iron, and CRP. In that study, multivariate logistic regression analysis found that Hb (β=-0.34), GDF-15 (β=-0.3), and hepcidin (β=-0.26) were predictors of anemia in patients with CKD [26]. Therefore, an increased GDF-15 level as well as iron deficiency might be useful markers for the screening of anemia, which is common in older adults.

Previous studies have examined GDF-15 determinants in community-dwelling older adults [9,27] and patients with mild renal impairment [28]. In all of these studies, Hb was an independent determinant of GDF-15. Lukaszyk et al. found that hepcidin was correlated with ferritin (r=0.65), iron (r=0.39), and TSAT (r=0.51) in patients younger than 65 years of age, and in those older than 65 years of age, hepcidin was only positively correlated with ferritin (r=0.7), but not with iron and TSAT [28]. This may be because the hepcidin level is determined by stored iron rather than serum iron, which may be associated with chronic inflammation due to aging. Our previous study of older women revealed that the log GDF-15 was determined by the log eGFR and Hb [9]. In our current study of community-dwelling older adults, we showed that the log GDF-15 may be determined by the hepcidin level in addition to eGFR and Hb.

The association between GDF-15 and hepcidin remains unclear. A significant association between GDF-15 and hepcidin has not been reported for patients with inflammatory and iron-deficiency anemia [11] or with CKD [26]. GDF-15 was reported to be positively correlated with hepcidin in non-anemic patients [29]. On the other hand, GDF-15 has been shown to inhibit hepcidin expression and then increase iron absorption regardless of iron overload in patients with beta-thalassemia [10]. In our study of community-dwelling older adults, GDF-15 was inversely correlated with hepcidin. Therefore, the differences in the correlations between GDF-15 and hepcidin may be accounted for by the various diseases of the study participants. Additional studies on the relationships between GDF-15 and hepcidin are needed.

The mechanisms involved in the effects of GDF-15 on anemia have been investigated in several studies [10,30]. In patients with anemia without inflammation, GDF-15 was produced and released in response to anemia or hypoxia. The increased GDF-15 level then led to a downregulation of hepcidin expression [10] and promoted iron absorption and iron recirculation in monocytes. Even in patients with anemia and inflammation, hepcidin expression was regulated by iron availability and associated with signals through bone morphogenic protein (BMP)-6 instead of inflammatory stimuli [30]. Our study showed that GDF-15 was not associated with inflammatory markers such as hs-CRP in community-dwelling older adults but was determined by the decrease in hepcidin levels as well as anemia and renal dysfunction.

This study has some strengths, such as the robust statistical analysis and the inclusion of multivariate regression analysis to explore complex relationships. However, it has some limitations. First, the sample size was not calculated because exercise class participants were included. Second, it has potential confounding factors or biases in participant selection, and the number of men was small. Therefore, further research is needed to clarify these issues. Research investigating the longitudinal associations between GDF-15, hepcidin, and Hb levels in older adults or exploring the therapeutic implications of targeting these pathways in the management of anemia and age-related diseases is furthermore needed.

## Conclusions

In community-dwelling older adults, the decreasing hemoglobin level was determined not only by a decrease in the serum iron level but also by an increase in the GDF-15 level. The increasing GDF-15 level was determined by a decrease in the hepcidin level as well as by anemia and renal dysfunction, and the decreasing hepcidin level was determined by a decrease in stored iron and a decrease in serum albumin level. Measurement of GDF-15 and hepcidin levels may be useful for risk stratification and treatment decisions in community-dwelling older adults. Due to the cross-sectional nature of the study design, further studies are needed to validate the utility of GDF-15 and hepcidin as biomarkers of anemia in older adults. Research investigating the longitudinal associations between GDF-15, hepcidin, and hemoglobin levels in older adults or exploring the therapeutic implications of targeting these pathways in the management of anemia and age-related diseases is needed in the future.
